# Non-suicidal self-injury motivation scale in a community sample of adolescents: a methodological study

**DOI:** 10.1186/s40359-024-01795-6

**Published:** 2024-05-24

**Authors:** Jungok Yu, Myo-Sung Kim, Miyoung Kim

**Affiliations:** 1 College of Nursing, Dong-A University, Busan, South Korea; 2 Department of Nursing, Dong-Eui University, Busan, South Korea; 3 Department of Nursing, Kaya University, Gimhae, South Korea

**Keywords:** Non-suicidal self-injury, Motivation, Adolescents

## Abstract

**Background:**

The prevalence of non-suicidal self-injury among South Korean adolescents has increased significantly, requiring academic attention. This methodological study aims to develop a non-suicidal self-injury motivation scale for adolescents and evaluate its validity and reliability.

**Methods:**

In the first phase of scale development, the factors constituting self-injury motivation were identified through a literature review and analysis of online counseling data from self-injuring adolescents. In the second phase, 45 initial preliminary items were derived based on the identified factors, and 38 preliminary items were selected through content validation by experts. In the scale validation phase, the survey was conducted using 38 items. Data were collected from adolescents with a history of self-injury, using exploratory factor analysis (EFA) involving 715 participants and confirmatory factor analysis (CFA) involving 537 participants. The EFA involved 27.0% male and 73.0% female participants, with a mean age of 16.83 years, and the CFA involved 20.7% male and 79.3% female participants, with a mean age of 16.15 years. The data collected were tested for validity and reliability using SPSS 28.0 and M-plus.

**Results:**

The EFA yielded four factors and 24 items. The factors were named interpersonal influence, emotion regulation, sensation seeking, and anti-suicide, and the scale had an explanatory power of 55.8%. In the CFA, the fit of the 23-item model after deleting one item with low standardized factor loadings was *x*^2^ = 1081.52 (*p* < .001), CFI = 0.829, RMSEA = 0.084, and SRMR = 0.075, confirming the acceptability of the self-injury motivation scale for adolescents. The scale evaluation results for convergent validity and discriminant validity met the criteria. The reliability test results showed that the overall reliability (Cronbach’s α) was 0.88, and the reliability (Cronbach’s α) of each factor was 0.89 for interpersonal influence, 0.83 for emotion regulation, 0.63 for sensation seeking, and 0.80 for anti-suicide, satisfying internal consistency.

**Conclusion:**

In this study, the self-injury motivation scale for adolescents in the community comprised four factors and 23 items. The scale can be used to examine self-injury motivation among adolescents in the community and to develop self-injury prevention intervention programs.

**Supplementary Information:**

The online version contains supplementary material available at 10.1186/s40359-024-01795-6.

## Background

Non-suicidal self-Injury (NSSI) is a person’s intentional and persistent harm to their body without the intention of attempting suicide [1]. While rates of non-suicidal self-injury (hereafter described as “self-injury” or “NSSI”) vary across studies, average prevalence of non-suicidal self-injury in adolescents was 16% [2]. Specifically, the prevalence of NSSI among South Korean adolescents has increased significantly [3].

Among adolescents, 80% of self-injurious behaviors involve cutting or stabbing the skin with a sharp object [4], suggesting that self-injurious behaviors may not be suicidal, but are likely to result in bodily harm. In addition, repeated self-injurious behaviors can easily lead to suicidal ideation and death [5, 6] and can cause psychological distress to friends and family members of self-injuring adolescents, making it a high-risk problem behavior among adolescents; therefore, the need to discuss prevention and intervention measures is urgent.

As self-injury often occurs during adolescence, when managing stress and tension and actively seeking alternative solutions is difficult, a better understanding of self-injury is necessary for prevention and intervention. Understanding how self-injury works is the starting point for helping people to stop it [7]; therefore, international efforts have been made to understand self-injury motivation from a variety of perspectives. In general, a large body of research reports that the most common motive for self-injurious behavior is its use as a coping mechanism to relieve emotional pain or discomfort; other explanations include self-punishment, attention seeking, and stimulation seeking [8]. However, the understanding of self-injury in adolescents and its functional characteristics is still lacking, especially in South Korea, where no systematic research investigating the motivations for self-injury among adolescents is available and valid and reliable functional assessments of NSSI appropriate for adolescents are needed.

Motivations for self-injury among South Korean adolescents have not been systematically studied, as studies on non-suicidal self-injury among Korean adolescents are few [4]. Regarding the self-injury measures used in each study, some risk factor exploration studies have simply examined the presence or absence of self-injurious behavior, either without using a scale or with a scale that only examines the presence or absence of self-injury and the characteristics of self-injury. For a more precise measure of non-suicidal self-injury, a scale that captures all relevant variables, such as motivation for self-injury as well as frequency and method of self-injury, is required. Accordingly, a recent domestic validation study used a foreign self-injury scale that includes a self-injury motivation measurement [9, 10]. As the study’s participants included university students and adults [9, 10], there are limitations in applying it to adolescents who are middle and high school students showing developmental characteristics that differ from those of adults. Despite the high prevalence of NSSI in adolescents, there have been insufficient scales to assess NSSI in adolescents, with most of the existing scales being developed in English-speaking countries [11]. Research is needed to develop a self-injury motivation scale for adolescents that can be adapted to conditions in South Korea and serve as a basis for further research.

Therefore, based on empirical data on NSSI behaviors among South Korean adolescents, this study aims to develop a scale that includes a measure of NSSI motivation in adolescents and to evaluate its reliability and validity.

## Methods

### Study design

The present study adopted an instrument development and validation design. This study was conducted in two phases: development and validation, following the scale development procedure of Devellis and Thorpe [12].

### Scale development procedure

#### Scale development phase

##### Identifying the conceptual framework and setting up the scale components

In the first phase of scale development, the self-injury motivation factors were identified through a literature review and analysis of online counseling data [3] from self-injuring adolescents. Literature research is the keywords of ‘Assessment, NSSI’, ‘Assessment, Self-injury’, ‘Assessment, Self-harm’ through RISS, KISS, KCI, DBpia, PsycINFO, and EBSCO. Among the self-harm scales identified in the above data, 11 instruments applied to adolescents were selected as the data for this study to identify the items of instruments. Through this process, the factors constituting adolescent self-injury motivation were identified, and a conceptual framework was established. This study defined NSSI in adolescents as intentionally damaging their own body without the intention to commit suicide, and self-injury motivation was defined as the purpose or reason for the self-injury behavior. Based on the literature review and analysis of self-injury counseling contents, this study classified factors associated with self-injury motivation in adolescents into intrapersonal and interpersonal factors [13]. In the non-suicidal self-injury function model, Nock and Prinstein [13] explain four functions for non-suicidal self-injury. However, it failed to repeatedly verify this in subsequent studies, and it was also classified as two factors in Korean studies. Intrapersonal factors refer to self-injury to control their internal emotional state, and interpersonal factors refer to self-injury to control their external situation. Refer to 13 individual functional scales in the Inventory of Statements About Self-injury (ISAS) [10] and analysis of online counseling data [3], intrapersonal motivations for self-injury include emotion regulation, gratification, self-punishment, anti-dissociation, anti-suicide, sensation seeking, and curiosity. Interpersonal motivations for self-injury include pain expression, attention seeking, interpersonal influence, blame avoidance, revenge, and peer bonding.

##### Organizing preliminary items

Based on the factors that constitute self-injury motivation identified in the literature review and analysis of self-injury counseling data, 45 initial items were developed. Most of the items were developed from the literature review and rephrased to reflect the actual experiences of the adolescents identified during the content analysis. The method of rating used the Likert scale, which is most commonly used when asking for responses to attitudes and perceptions. Each of the NSSI motivation items was scored on a scale from 0 points for “not relevant at all,” 1 point for “somewhat relevant,” and 2 points for “very relevant.” In addition to self-injury motivation, the scale included 15 items assessing methods and frequency of self-injury and 8 items on self-injury-related characteristics such as timing, duration, and treatment.

##### Content validity evaluation by experts

An expert group comprising four professors of psychiatric nursing, two psychiatrists, one clinical psychotherapist, four school nurses, and one counselor was organized for the content validity evaluation of the preliminary self-injury motivation scale. In September 2021, the questionnaire was distributed to facilitate a content validity evaluation of 45 preliminary items on self-injury motivation. Experts judged the content validity based on an item-level content validity index (I-CVI) to identify items with an inter-rater agreement of 80% or higher. An item readability assessment was conducted to ensure that the items in the self-injury motivation scale were easy for adolescents to read and that the wording was appropriate. The readability assessment was conducted by a single expert in Korean literature. After the experts’ content validity evaluation on 45 items, 38 items had an I-CVI of 80% or higher, and seven items were removed; these were removed to avoid redundancy.

#### Scale validation phase

##### Participants

This study’s participants were between 14 and 18 years old, the age of middle and high school students in South Korea, and experienced NSSI, which referred to the intentional harming of their own body without intending to commit suicide.

While the issue of sample size in factor analysis has many different views and heuristic rules, the traditional criteria for a minimum sample size in factor analysis can be divided into two categories. The first is a criterion for absolute sample size. Based on the literature, a sample size of 100 is inadequate, 200 is adequate, 300 is good, 500 is very good, and 1,000 or more is excellent. The second criterion is the N: P ratio (sample size required for factor analysis), which has been variously suggested as 20:1, 10:1, and 6:1 [14].

Therefore, considering both the absolute sample size and the N: P ratio, this study adopted 20:1 as the sample size required for factor analysis, to ensure sufficient stability of the statistical test. As the EFA involved 38 preliminary items, it was intended to collect data from 760 participants. While 759 participants were recruited for the exploratory factor analysis, 44 non-respondents were excluded, leaving 715 participants in total for the analysis. For the CFA, the sample size was 480, which was required for the 24-item NSSI motivation scale, and 537 participants were recruited with no missing data or outliers; therefore, 537 participants in total were included in the analysis.

Table 1 shows the general characteristics of participants who participated in the evaluation of the validity and reliability of the preliminary scale developed in this study. The EFA involved 27.0% male and 73.0% female participants, with a mean age of 16.83 years, and the CFA involved 20.7% male and 79.3% female participants, with a mean age of 16.15 years.

**Table 1:** Characteristics of participants

Variables	Categories	EFA *n*(%) *n* = 715	CFA *n*(%) *n* = 537
Gender	Male	193 (27.0)	111 (20.7)
	Female	522 (73.0)	426 (79.3)
Age	M ± SD	16.83 ± 1.31	16.15 ± 1.35
	14	154 (21.5)	65 (12.1)
	15	142 (19.9)	144 (26.8)
	16	151 (21.1)	93 (17.3)
	17	199 (27.8)	114 (21.2)
	18	69 (9.7)	121 (22.5)
Academic grade	High	59 ( 8.3)	43 (8.0)
	Mid-high	182 (25.5)	129 (24.0)
	Middle	209 (29.2)	161 (30.0)
	Mid-low	183 (25.6)	148 (27.6)
	Low	82 (11.5)	56 (10.4)
Economic level of a family	High	26 (3.6)	28 (5.2)
	Mid-high	154 (21.5)	106 (19.7)
	Middle	340 (47.6)	246 (45.8)
	Mid-low	159 (22.2)	129 (24.0)
	Low	36 (5.0)	28 (5.2)

Table 2 shows the self-injury-related characteristics of the participants in this study. For participants in the EFA, the mean age at onset of self-injury was 13.79 ± 2.03 years and the mean duration of self-injury was 12.61 ± 18.08 months. The most frequent method of self-injury was “hitting oneself with a hand or tool,” at 55.4%. For participants in the CFA, the mean age at onset of self-injury was 13.64 ± 1.97 years, and the mean duration of self-injury was 14.89 ± 18.10 months. The most frequent method of self-injury was, again, “hitting oneself with a hand or tool,” at 73.9%.

**Table 2:** Self-injury-related characteristics of participants

Self-injury-related characteristics	EFA *n*(%) or M ± SD *n* = 715		CFA *n*(%) or M ± SD *n* = 573	
	yes	no	yes	no
*Age of onset of NSSI (years)*	13.79 ± 2.03		13.64 ± 1.97	
*Duration of NSSI (months)*	12.61 ± 18.08		14.89 ± 18.10	
*Methods of NSSI*				
Cutting oneself with a sharp object	241 (33.7)	474 (66.3)	263 (49.0)	274 (51.0)
Hitting oneself with a hand or tool	396 (55.4)	319 (44.6)	397 (73.9)	140 (26.1)
Biting oneself	242 (33.8)	473 (66.2)	279 (52.0)	258 (48.0)
Pinching oneself to the point where it hurts	252 (35.2)	463(64.8)	278(51.8)	259(48.2)
Scratching normal skin to form a wound	268 (37.5)	447 (62.5)	323 (60.1)	214 (39.9)
Picking or pulling one’s own hair to the point of pain	229 (32.0)	486 (68.0)	250 (46.6)	287 (53.4)
Puncturing the skin or under the nail with a pointed tool	118 (16.5)	597 (83.5)	153 (28.5)	384 (71.5)
Bashing oneself in the head	232 (32.4)	483 (67.6)	251 (46.7)	286 (53.3)
Hitting a wall until one’s own hand is bruised	165 (23.1)	550 (76.9)	198 (36.9)	339 (63.1)
Preventing wounds on one’s own body from healing	143 (20.0)	572 (80.0)	153 (28.5)	384 (71.5)
Overdosing on drugs	82 (11.5)	633 (88.5)	77 (14.3)	460 (85.7)
Starving oneself (with the intention of hurting oneself)	338 (47.3)	377 (52.7)	154 (28.7)	383 (71.3)
Burning oneself	36 (5.0)	679 (95.0)	29 (5.4)	508 (94.6)
Strangling oneself	159 (22.2)	556 (77.8)	175 (32.6)	362 (67.4)
Other	6 (0.8)	709 (99.2)	2 (0.4)	535 (99.6)
*Characteristics related to the severity of NSSI*				
Did you have negative feelings or thoughts immediately before the self-injurious behavior?	549 (76.8)	166 (23.2)	490 (91.2)	47 (8.8)
Were there any relationship difficulties or problems with others immediately before the self-injurious behavior?	435 (60.8)	280 (39.2)	400 (74.5)	137 (25.5)
Before injuring yourself, did you have any hard-to-resist urges or desires to hurt yourself?	343 (48.0)	372 (92.0)	353 (65.7)	184 (34.3)
Do you often think about self-injuring, even when you are not doing it?	231 (32.3)	484 (67.7)	212 (39.5)	325 (60.5)
Is the self-injurious behavior causing a significant inconvenience or problem in school, interpersonal relationships, or daily life?	115 (16.1)	600(83.9)	109 (20.3)	428 (79.7)
Have you ever been treated or hospitalized for self-inflicted injuries?	45 (6.3)	670 (93.7)	50 (9.3)	487 (90.7)

##### Data collection period and methods

As construct validity and model fit are exaggerated if the participants of EFA and CFA are the same [15], EFA was conducted after the first data collection, and CFA was conducted after the second data collection with different participants.

Recruitment was conducted by Macromill Embrain, an online survey agency. Primary data collection was conducted from March 17 to March 22, 2022. Secondary data collection was conducted from July 19 to July 27, 2022, and recruitment was conducted after excluding the adolescents in the online panel who were also included in the primary data collection.

As the recruitment and survey participation were conducted online, the survey was conducted in compliance with ethical guidelines, with the consent of both the participants and their parents or legal guardians, and their privacy was strictly maintained. The participants were provided with an information sheet and informed consent and were invited to complete online questionnaires, which were distributed through online survey tool.

##### Data analysis

The statistical programs SPSS 27.0 and Mplus 8.0 were used. The participants’ general characteristics were analyzed using descriptive statistics of frequencies and percentages. For item analysis, each item’s mean, standard deviation, skewness, and kurtosis were examined, and the goodness-of-fit of the items was analyzed through inter-item correlation and item-total correlation.

An EFA was conducted to provide evidence for the construct validity of the preliminary scale. Kaiser-Meyer-Olkin (KMO) values were checked, and Bartlett’s test of sphericity was performed to determine the adequacy of the data for the exploratory factor analysis. In the EFA based on common factor analysis, factors were extracted using principal axis factoring, and squared multiple correlation (SMC) was used as the initial value of common variance (communality). To determine the number of factors in the exploratory factor analysis, the scree plot and the cumulative explained variance ratio were used. The final factor loadings of the items on each factor were determined using oblique rotation, which allowed for correlation between factors, and the promax method. In the promax method, a ${\kappa }$ (kappa) index determined how much correlation between factors was allowed and ${\kappa }=4,$ recommended by Hendrickson and White [16], was followed.

A CFA was conducted to verify the construct validity. In CFA, goodness-of-fit indices were evaluated by chi square statistic, standardized root mean residual (SRMR), root mean square error of approximation (RMSEA), and comparative fit index (CFI). The reference values of the goodness-of-fit index are SRMR of 0.08 or less, RMSEA of 0.10 or less, and CFI of 0.90 or more.

##### Ethical considerations

The content and methods of this study were approved by the Institutional Review Board (IRB) of D University. Approvals were received for the development of the scale, an EFA study (202,112-HR-084-04), and a CFA study (202,205-HR-025-04). At the time of the survey, the purpose and method of the study, study procedures and methods, time required for the survey, and matters related to personal information and withdrawal from the study were explained, and the survey participants voluntarily agreed to participate. As the survey was conducted through an external specialized survey agency, the participants’ responses were automatically coded and stored in an Excel file, and the principal investigator received a separate password-secured Excel file from the company via email with no personally identifiable information collected during the data collection process. The external survey agency was also certified for its good privacy protection. The excel file will be deleted three years after the end of the study in accordance with the Personal Information Protection Act.

## Results

### Item analysis

After evaluating the mean, standard deviation, skewness, and kurtosis for each of the 38 self-injury preliminary items, the mean scores for the items, on a 3-point scale, ranged from 1.08 to 2.00, with standard deviations from 0.33 to 0.81. Items with means close to 1 (completely irrelevant) were observed (e.g., items 20, 27, 28, 32, and 36), and the absolute values of skewness and kurtosis for items 20, 27, 28, 32, and 36, with problems regarding the mean and standard deviation, did not meet Kline’s [17] criteria for normality.

Thereafter, the inter-item correlation was analyzed for the overall scale. After evaluating the correlation between the items through the correlation matrix between the items, the correlations were all positive, indicating no problem, except for item 38. Item 38 was negatively correlated with items 1 (-0.025), 3 (-0.031), and 34 (-0.009) and had generally low positive correlations with the rest of the items. No overly high correlations that might suggest redundant questions were observed.

The items were analyzed using Cronbach’s ${\alpha }$, the reliability of the scale, as well as the corrected item-total correlations (discrimination) and Cronbach’s ${\alpha }$ after item removal. The reliability (${\alpha }$) of all 38 items was 0.920, indicating a very high reliability of internal consistency. After examining the corrected item-total correlation, the most problematic item was item 38, which had a discrimination of 0.19, requiring removal or complete revision. Item 26 with a score of 0.253 was identified as needing improvement. After analyzing the Cronbach’s alpha value after item removal, the reliability of the scale increased after removing item 38, compared to that of the original scale (38 items), at 0.920. The analysis of the change in discrimination and reliability with the remaining 37 items, after removing item 38, revealed that the discrimination of item 26 was 0.234, indicating that it still required revision or improvement. The Cronbach’s alpha after removing the item was 0.923, which was slightly higher than the reliability of the original scale (37 items) at 0.922. Finally, after analyzing the change in discrimination and reliability with the remaining 36 items after removing item 26, no item was problematic.

Based on the results of the item analysis so far, items 20 (to offend others), 27 (to imitate those you admire, such as TV celebrities, and want to be like them), 28 (to feel like a member of a group), 32 (to express friendship and bonding with friends and loved ones), and 36 (to fit in with friends and others) were identified as problematic through the descriptive statistics. Item 38 (for no particular reason) identified as problematic through the analysis of reliability and correlation; 26 (to try it out to see what it is like) with insufficient discrimination were removed after reviewing their content.

### Validity analysis

#### Construct validity evaluation: EFA

An EFA was conducted on 31 items, excluding the seven items mentioned above, to confirm the factor structure. Prior to starting the analysis, the KMO measure of sampling adequacy was examined, and Bartlett’s test of sphericity was conducted to ensure that the data collected was suitable for factor analysis. The KMO sample fit of the data in this study was 0.927, indicating their suitability for factor analysis [18], and the chi-square approximation of Bartlett’s test of sphericity was 9004.92, which was statistically significant at p < 0.001, suggesting sufficient correlation between the items as data suitable for factor analysis.

While there was some ambiguity in how to determine the number of factors in a scree plot as a pictorial method, four or six factors were considered adequate. In fact, after extracting factors based on eigenvalues of 1 or more, which was another way to determine the number of factors, six factors were extracted; however, factor structure indicated by the factor loadings and the structure of the factors in the pattern matrix was unclear and difficult to interpret. Therefore, a four-factor structure seemed appropriate.

The final factor loadings of the items on each factor were determined using the pattern matrix of factor loadings with a promax rotation to determine how the items formed the dimensions. First, items that did not have sufficient factor loadings were screened and removed for each factor. Items 2, 4, 12, 31, and 37 were removed, as they did not meet the criteria. After performing a secondary EFA on the 26 remaining items after removal, items 8 and 35, with factor loadings below 0.4, under the four-factor structure were removed. As a result, 24 items were finally selected. Table 3 shows the pattern matrix results. The cumulative explained variance ratio of the four factors was 55.8%.

**Table 3:** Result of exploratory factor analysis *N* = 715

Item		Factor 1	Factor 2	Factor 3	Factor 4
7	To get more attention from parents	0.601			
11	To get attention from others, such as friends or teachers	0.585			
15	To change the way people treat you	0.651			
16	To hurt someone close to you	0.614			
19	To make your parents understand you better	0.721			
23	To get help or care from other people	0.709			
24	To take revenge on someone	0.535			
30	To show others how hurt you are	0.733			
34	To let others know you are struggling	0.715			
1	To release anger		0.449		
3	To express the pain in your heart		0.421		
5	To alleviate the frustration		0.654		
6	Because a sick body is better than a sick mind		0.496		
9	To reduce feelings of sadness or depression		0.489		
13	To reduce anxiety and tension		0.515		
21	To feel better (relieved)		0.515		
25	To punish yourself		0.676		
29	Because you hate and are dissatisfied with yourself		0.865		
33	To express your anger at yourself for being useless and stupid		0.876		
17	To make yourself comfortable			0.512	
18	To feel a sense of excitement			0.648	
22	To feel a sense of satisfaction or accomplishment			0.722	
10	To stop thinking about suicide				0.876
14	To avoid the temptation of attempting suicide				0.647
Eigenvalue		7.372	3.217	1.573	1.236
Explained variance (%)		30.715	13.404	6.554	5.151
Total explained variance (%)		30.715	44.120	50.674	55.824
Cronbach’s ${\alpha }$		0.877	0.868	0.699	0.763
Kaiser-Meyer-Olkin measure = 0.917					
Bartlett’s test of sphericity X^2^ = 6988.001, p < 0.001					

After determining the actual meaning of each factor by considering the content of the items based on the size of the factor loadings, Factors 1, 2, 3, and 4 were interpersonal influence, emotion regulation, sensation seeking, and anti-suicide, respectively.

#### Construct validity evaluation: CFA

A CFA was conducted to verify the suitability of the structure of the 24 self-injury motivation items of the four factors derived from the EFA, and the data of 537 participants were analyzed. The means and standard deviations of all 24 variables used as indicator variables in the CFA model were within acceptable ranges, with no outliers observed. After estimating skewness and kurtosis, skewness ranged from − 0.141 to 2.843, and kurtosis ranged from − 1.494 to 7.300. After estimating the CFA model, the null hypothesis that the model fits the data with x^2^ = 1120.97 was rejected at the $\text{p}<0.001$ level. The x^2^ model fit test determines whether the model fits the data perfectly; therefore, test rejection only means that the model does not fit the data perfectly without ruling out the possibility that the model approximates the data. As is well known, most x^2^ tests, including the goodness-of-fit test, are sensitive to sample size. Therefore, it is standard practice to check a range of approximate goodness-of-fit indices to determine the overall fit of the model. First, the CFI was 0.828, indicating a poor fit according to Bentler’s [19] criterion of 0.90 or more. However, a simulation study by Kenny and McCoach [20] demonstrated that, even in a correctly specified model, the CFI tends to be underestimated when the number of observed variables is too large. For this reason, if other model-fit indices are good but only CFI is inadequate as the number of indicator variables in a scale development and validation study increases, then this may be considered a characteristic of the CFI index. The RMSEA was 0.081, which was close to Browne and Cudeck’s [21] criterion of a good fit of 0.08 or less and was not over 0.10, which Kline [17] reported as not a bad fit. SRMR was 0.073, well within Hu and Bentler’s [22] criterion of 0.08 or less. After examining the CFA model and its model fit, it showed a good fit in the context of scale development and validation.

While several methods have been proposed to examine convergence validity, the most popular method entails checking the standardized factor loadings. No single value determines what a standardized factor-loading estimate should be, as it varies from discipline to discipline and situation to situation; however, Kline [17] suggested a value of 0.7 or higher. This value is very conservative and idealized and very difficult to achieve in real-world situations using any factor-loading estimate. Hair, Black, Babin, and Anderson [15] found that a value greater than 0.5 was an acceptable standardized factor loading value, and Wang and Wang [23] and Stevens [24] considered a value of 0.4 or greater as acceptable for convergence. Most of the standardized factor-loading estimates were between 0.415 and 0.847, suggesting that the indicator variables were generally good measures of the construct, satisfying convergence validity. However, the first indicator variable measuring regulation, Regulation1, had a standardized factor loading of 0.344, which did not meet even the most lenient criteria of Wang and Wang [23] and Stevens [24]. Therefore, without further checking the convergent or discriminant validity, item 1 was removed, and the CFA was performed again.

Table 4 provides the goodness-of-fit indices for the modified CFA model. The results of estimating the measurement model showed that the overall fit was almost the same as that of the CFA model estimated earlier. Table 4 shows factor loading estimates for convergent and discriminant validity of the factor structure, and Table 5 shows inter-factor correlation coefficient estimates for discriminant validity. The standardized factor loadings provided in Table 4 ranged from 0.413 to 0.847, with most of the values above 0.5, which satisfied the criteria of Hair et al. [15], and those of items 24, 3, 25, and 18 were from 0.4 to 0.5, which satisfied the criteria of Wang and Wang [23] and Stevens [24]. While this was not a very highly standardized factor-loading estimate, the overall convergence validity was satisfactory. Next, there are several ways to check for discriminant validity, the most widely used being that the correlation coefficients between factors should not be too high. Kline [17] and Voorhees et al. [25] suggested that inter-factor correlation coefficients should not exceed 0.9 to ensure discriminant validity, while Rönkkö and Cho [26] argued for values below 0.8. The correlations between interpersonal influence, emotion regulation, sensation seeking, and anti-suicide were no higher than 0.690, ensuring discriminant validity by any criteria.

**Table 4:** Final result of confirmatory factor analysis *N* = 537

Factor	Item	Standardized estimates	S.E	Z	Estimates
Interpersonal influence	7	1.000	-	-	0.706
	11	0.947	0.060	15.881	0.739
	15	0.925	0.062	14.985	0.694
	16	0.633	0.053	11.897	0.541
	19	0.893	0.055	16.273	0.728
	23	1.101	0.065	17.007	0.793
	24	0.425	0.047	9.066	0.415
	30	0.969	0.061	15.910	0.744
	34	1.257	0.073	17.109	0.804
Emotional regulation	3	1.678	0.258	6.512	0.482
	5	2.269	0.316	7.177	0.649
	6	2.273	0.328	6.939	0.590
	9	2.172	0.304	7.138	0.639
	13	2.069	0.295	7.004	0.600
	21	2.116	0.302	7.018	0.600
	25	1.567	0.246	6.366	0.457
	29	2.305	0.321	7.171	0.656
	33	2.348	0.326	7.196	0.658
Sensation seeking	17	1.000	-	-	0.711
	18	0.416	0.074	5.646	0.413
	22	0.842	0.111	7.576	0.657
Anti-suicide	10	1.000	-	-	0.797
	14	1.175	0.085	13.788	0.847
Model fitness: χ2 = 1081.52(ρ < 0.001), CFI = 0.829, RMSEA (95%CI) = 0.084(0.079- 0.090), SRMR = 0.075					

CFI, Comparative fit index; RMSEA, Root mean square error of approximation; SRMR, Standardized root mean residual

**Table 5:** Correlation coefficients between factors in the final confirmatory factor analysis model

	Factor 1	Factor 2	Factor 3	Factor 4
Interpersonal influence	1.000			
Emotional regulation	0.283	1.000		
Sensation seeking	0.303	0.690	1.000	
Anti-suicide	0.257	0.627	0.485	1.000

#### Reliability

Cronbach’s α and McDonald’s omega (ω) were obtained to verify the reliability of the self-injury motivation scale for adolescents in this study. The Cronbach’s α for the overall scale was 0.88, and McDonald’s omega (ω) was 0.84. The Cronbach’s α of each factor was 0.89 for interpersonal influence, 0.83 for emotion regulation, 0.63 for sensation seeking, and 0.80 for anti-suicide, satisfying internal consistency.

## Discussion

This study extracted attributes of self-injury motivation through a literature review and analysis of the counseling data contents for self-injuring adolescents, developed a self-injury motivation scale suitable for adolescents in the community, and verified its validity and reliability. Based on this study’s results, self-injury motivation among adolescents in the community comprised four factors: interpersonal influence, emotion regulation, sensation seeking, and anti-suicide, for 23 items in total (supplementary file 1).

The first factor of self-injury motivation identified in this study, “interpersonal influence,” refers to gaining support and attention from parents or others or manipulating relationships to gain a desired advantage. Additionally, interpersonal influence includes wanting to let others know one is hurting or struggling and taking revenge on others. In this study, the “interpersonal influence” factor had an Eigenvalue of 7.372 and an explanatory power of 30.7%, having the highest explanatory power among all the factors in the scale. This result conflicted that of the study by Swannell et al. [27] which identified the factors of the Self-Injury Motivation Scale (SIMS-A) in adolescent inpatients, where “emotion regulation” was the factor with the highest explanatory power. In their functional model of NSSI, Nock & Prinstein [13] described four main motivations for NSSI, They categorized the intrapersonal motivations into positive reinforcement, in which a person sought to gain attention and support from parents or others through NSSI, and negative reinforcement, in which a person sought to avoid obligations or responsibilities in social situations and interpersonal relationships or to escape from unwanted situations through NSSI. However, for adolescents in the community who participated in this study, items related to responsibility avoidance, such as “to prevent others from being angry with me” or “to get out of doing something I don’t want to do,” which corresponded to negative social reinforcement in the initial questionnaire, were removed during exploratory factor analysis. In addition, all items related to peer bonding (e.g., to fit in with friends and others) were removed, resulting in a social factor that is different from Functional Assessment of Self-Mutilation (FASM) [9] and Ottawa Self-injury inventory (OSI ) [28] and similar to the “communicating with/influencing others” factor of SIMS-A [27].

The second factor of self-injury motivation identified in this study is “emotion regulation,” which refers to the use of NSSI to relieve unpleasant emotions such as anger or frustration. According to the functional model of NSSI, Nock & Prinstein [13] suggest that self-injury for intrapersonal motivations can also be categorized into negative reinforcement, which aims to eliminate or relieve negative thoughts and feelings, and positive reinforcement, which aims to activate a desired stimulus. In this study, the reasons for reducing anger, anxiety, frustration, depressed mood, and dislike of oneself, which fall under the category of negative reinforcement, mainly constituted the second factor, “emotion regulation.” In the Korean validation study of the FASM [9], using a self-injury motivation scale based on Nock & Prinstein’s [13] functional model of suicidal self-injury, two factors of interpersonal motivation and intrapersonal motivation were identified instead of four factors, and intrapersonal motivation included both positive reinforcement and negative reinforcement items.

Conversely, the third factor of self-injury motivation identified in this study is “sensation seeking,” which is classified as a separate factor of intrapersonal motivations for NSSI, such as NSSI to achieve excitement or pleasure. In OSI [28], three of the four factors constituting self-injury motivation were related to intrapersonal factors, which was in line with the separation of “sensation seeking” as a separate factor in this study. This seemed to reflect the demographic characteristics of the adolescent group.

The fourth factor of self-injury motivation identified in this study is “anti-suicide,” which refers to the desire to stop thoughts and urges of suicide through NSSI. This factor was absent from FASM [9], included as a component of “emotion regulation” in OSI [28], and a single item and as a component of “psychosea/lack of insight” in SIMS-A [27]. South Korea has the highest suicide rate in the OECD, with 2020 statistics showing a rate of 24.1 suicides per 100,000 people, more than double the OECD average of 11.1 [29]. The suicide rate among South Korean adolescents has been steadily increasing from 4.2 per 100,000 in 2015 to 7.1 in 2021 [29], and by cause of death, “intentional self-injury (suicide)” accounts for the largest proportion of deaths among those aged 10–19 (43.7%, Cause of Death Statistics by Statistics Korea). In this context, the fourth factor, “anti-suicide,” is considered an important factor in South Korea.

The components of NSSI may vary by cultural background or between adults and adolescents [11]. Existing scales developed in English-speaking countries may have limitations in measuring non-suicidal self-injury motivation among South Korean adolescents. The development of this scale was significant in that there was a need to specifically identify the current status of NSSI in the community, given the recent increase in the number of adolescents engaging in NSSI in South Korea [3].

Nevertheless, there were some limitations in this study. While this study’s participants were adolescents, out-of-school adolescents were not included. However, since South Korea has an extremely low rate of out-of-school adolescents (2.6%), this study may have reflected the characteristics of adolescents in general. This study also could not validate the scale with adolescents in psychiatric hospitals. Future validation studies will be required to determine if the same factors are identified in adolescents admitted to psychiatric hospitals.

## Conclusion

This study was conducted to develop a non-suicidal self-injury motivation scale for adolescents in the community and evaluate its reliability and validity. The tool development process revised 45 items to 31 items through content validity evaluation and item analysis and finalized four factors and 23 items through exploratory and confirmatory factor analyses. Each of the NSSI motivation items was scored on a scale from 0 points for “not relevant at all,” 1 point for “somewhat relevant,” and 2 points for “very relevant.” The finalized scale was highly reliable, with construct validity ensured.

This study will provide a community-based assessment of the prevalence of NSSI among adolescents and serve as a basis for developing interventions to prevent the rapidly increasing prevalence of NSSI among adolescents.

### Electronic supplementary material

Below is the link to the electronic supplementary material.

Supplementary Material 1

## Data Availability

The datasets used and/or analysed during the current study are available from the first author on reasonable request.

## Abbreviations

EFA: Exploratory factor analysis
CFA: Confirmatory factor analysis
Hereafter described as “self-injury” or “NSSI”: Non-suicidal self-injury
I-CVI: Item-level content validity index
KMO: Kaiser-Meyer-Olkin
SMC: Squared Multiple Correlation
SRMR: Standardized root mean residual
RMSEA: Root mean square error of approximation
CFI: Comparative Fit Index
IRB: Institutional Review Board
SIMS-A: Self-Injury Motivation Scale
FASM: Functional Assessment of Self-Mutilation
OSI: Ottawa Self-injury inventory
