# Supplementary material for: Non-suicidal self-injury motivation scale in a community sample of adolescents: a methodological study
Source: BMC Psychol. 2024 May 24;12:292. doi: 10.1186/s40359-024-01795-6 (PMC11127424; doi:10.1186/s40359-024-01795-6)
Supplement: Supplementary file 1 — Supplementary Material 1 [file 40359_2024_1795_MOESM1_ESM.docx]

1. Over the past year (12 months), have you done any of the things stated below to intentionally harm your body without the intent to kill yourself? If no, check the box for 0; if yes, check the box that indicates the frequency (number of days) of the behavior.

| **Number** | **Ways of self-harm** | **Frequency of self-harm (Number of days)** | | | | | |
| --- | --- | --- | --- | --- | --- | --- | --- |
|  |  | **0** | **1 day** | **2 days** | **3 days** | **4 days** | **≥ 5 days** |
| 1 | Scratching your body with sharp objects to cause injuries. |  |  |  |  |  |  |
| 2 | Hitting your body with your hands or tools. |  |  |  |  |  |  |
| 3 | Biting your body parts to the extent that it causes injuries. |  |  |  |  |  |  |
| 4 | Pinching your body parts to the extent that it causes injuries. |  |  |  |  |  |  |
| 5 | Scratching your normal skin until it bleeds. |  |  |  |  |  |  |
| 6 | Yanking or pulling your hair to the point of pain. |  |  |  |  |  |  |
| 7 | Using sharp objects to poke beneath your nails or skin. |  |  |  |  |  |  |
| 8 | Forcefully bumping your head. |  |  |  |  |  |  |
| 9 | Hitting walls to the extent that your hands are injured. |  |  |  |  |  |  |
| 10 | Preventing wounds on your body from healing. |  |  |  |  |  |  |
| 11 | Overdosing on drugs. |  |  |  |  |  |  |
| 12 | Intentionally starving. |  |  |  |  |  |  |
| 13 | Burning your skin. |  |  |  |  |  |  |
| 14 | Strangling yourself. |  |  |  |  |  |  |
| 15 | Others ( ) |  |  |  |  |  |  |

2. If you have harmed yourself,

2-1. At what age did you first harm yourself? ______years

2-2. How long have you been harming yourself? _____years ______months

2-3. Over the past year (12 months),

| 1 | Have you had negative emotions or thoughts (such as sadness, anxiety, tension, distress, self-criticism) immediately before harming yourself? | Yes | No |
| --- | --- | --- | --- |
| 2 | Have you had difficulties or problems with relationships with other people immediately before harming yourself? | Yes | No |
| 3 | Have you experienced irresistible impulses or desires to hurt yourself before harming yourself? | Yes | No |
| 4 | Do you often think about harming yourself even when you are not engaging in self-harming behavior? | Yes | No |
| 5 | Do your self-harm behaviors seriously interfere or cause problems with your studies, interpersonal relationships, or daily life? | Yes | No |
| 6 | Have you received medical treatment or been hospitalized because of self-inflicted injuries? | Yes | No |

3. Please choose the number that best matches your thoughts regarding the **reason** for intentionally harming your body without the intent to kill yourself.

| **Item** | **Not related at all** | **Somewhat related** | **Strongly related** |
| --- | --- | --- | --- |
|  | **0** | **1** | **2** |
| To express that my heart is in pain |  |  |  |
| To relieve frustration |  |  |  |
| Because physical pain is better than emotional pain |  |  |  |
| To get more attention from my parents |  |  |  |
| To lessen sad or depressed mood |  |  |  |
| To stop thinking about suicide |  |  |  |
| To get attention from other people, like friends and teacher |  |  |  |
| To lessen anxiety and tension |  |  |  |
| To avoid the impulse to attempt suicide |  |  |  |
| To change how people treat me |  |  |  |
| To hurt my loved one |  |  |  |
| To feel comfort |  |  |  |
| To experience a thrilling sense of excitement |  |  |  |
| To make my parents understand me better |  |  |  |
| Because it makes me feel relieved |  |  |  |
| To feel satisfaction or a sense of achievement |  |  |  |
| Because I want to get help or care from other people |  |  |  |
| To seek revenge on others |  |  |  |
| To punish myself |  |  |  |
| Because I am dissatisfied and hate myself |  |  |  |
| To show others how hurt I am |  |  |  |
| To express anger toward my useless and foolish self |  |  |  |
| To let others know that I’m having a hard time |  |  |  |
